# Theoretical Investigation on H_2_O_2_-Ng (He, Ne, Ar, Kr, Xe, and Rn) Complexes Suitable for Stereodynamics: Interactions and Thermal Chiral Rate Consequences

**DOI:** 10.3389/fchem.2018.00671

**Published:** 2019-01-18

**Authors:** Yuri Alves de Oliveira Só, Pedro Henrique de Oliveira Neto, Luiz Guilherme Machado de Macedo, Ricardo Gargano

**Affiliations:** ^1^Institute of Physics, University of Brasília, Brasília, Brazil; ^2^Institute of Biological Sciences, Faculty of Biotechnology, Federal University of Pará, Belém, Brazil

**Keywords:** hydrogen peroxide, noble gases, stereodynamics, chirality, thermal chiral rate, enantiomers, TST method, AIM theory

## Abstract

Although molecular collisions of noble gases (Ng) can be theoretically used to distinguish between the enantiomers of hydrogen peroxide - H_2_O_2_ (HP), little is known about the effects of HP-Ng interactions on the chiral rate. In this work, the chiral rate as a function of temperature (CRT) between enantiomeric conformations of HP and Ng (Ng=He, Ne, Ar, Kr, Xe, and Rn) are presented at MP2(full)/aug-cc-pVTZ level of theory through a fully basis set superposition error (BSSE) corrected potential energy surface. The results show that: (a) the CRT is highly affected even at a small decrease in the height of trans-barrier; (b) its smallest values occur with Ne for all temperatures between 100 and 4,000 K; (c) that the decrease of CRT shows an inverse correlation with respect to the average valence electron energy of the Ng and (d) Ne and He may be the noble gases more suitable for study the oriented collision dynamics of HP. In addition to binding energies, the electron density *ρ* and its Laplacian ∇^2^*ρ* topological analyses were also performed within the atoms in molecules (AIM) theory in order to determine the nature of the HP-Ng interactions. The results of this work provide a more complete foundation on experiments to study HP's chirality using Ng in crossed molecular beams without a light source.

## 1. Introduction

Hydrogen peroxide - H_2_O_2_ (HP) is a molecule of interest in a large and diverse number of fields in addition to its industrial uses. For example, it has emerged as a major metabolite in redox signaling and regulation (Antunes and Brito, 2017; Sies, 2017), and its presence was observed in Martian atmosphere (Encrenaz et al., 2004) and also on the surface of Jupiter's moon Europa (Carlson et al., 1999). The HP is interesting since it is simplest molecule that exhibits internal (torsional) rotation and chirality. Furthermore, this molecule can form dimers (Dobado and Molina, 1993; González et al., 1997), clusters (Yu and Yang, 2011), complexes with water (Mo et al., 1994; González et al., 1997) and with biologically important molecules such as adenine (Dobado and Molina, 1999), DNA (Piatnytskyi et al., 2016), glycine (Shi and Zhou, 2004) or nitrosamines (Roohi et al., 2010). These features indicate that HP should be a better proton donor for hydrogen bonding than water. Thus, the understanding of how the relative orientation of the O-H can lead to a weakly complex or a chemical reaction has also been paid considerable attention due to its implication in atmospheric chemistry and oxidation reactions (Lundell et al., 1998, 2001; Daza et al., 2000; Goebel et al., 2000, 2001a,b, 2002; Molina et al., 2002; Pehkonen et al., 2004; Mucha and Mielke, 2009; Grzechnik et al., 2013). Moreover, HP's properties have been investigated, such as its isolated chirality (Roncaratti and Aquilanti, 2010), stereomutation (Fehrensen et al., 2007; Bitencourt et al., 2008), size-dimensional wave packets (Wang et al., 2012), spectroscopy (Hunt et al., 1965; Małyszek and Koput, 2013; Al-Refaie et al., 2015) and rotation barriers (Song et al., 2005).

On the other hand, the hydrogen peroxide seems to be a prototypical model to be used into experiments to observe chirality in crossed molecular beam without a light source (Palazzetti et al., 2013), a frontier in research of stereodynamics which is still at early stages (Su et al., 2013; Lombardi and Palazzetti, 2018). In these kind of experiments, the molecular orientation control on the intense continuous beam is mandatory to the phenomena of chiral selectivity to be demonstrated (Aquilanti et al., 2005). For this reason, the interaction between HP and atoms, molecules and ions is so relevant to sterodynamics studies (Barreto et al., 2007, 2010; Lombardi et al., 2011; Roncaratti et al., 2014; Leal et al., 2016).

In the present paper we investigated the dynamics of the chiral molecule HP interacting by van der Waals forces with noble gases Ng (Ng=He, Ne, Ar, Kr, Xe and Rn) in order to obtain the chiral rate as a function of temperature (CRT) for these complexes, as well as its consequences for the chiral kinetic interconversion when tunneling effect is included. The aim of this work was to understand how the kinetic interconversion of the two HP's isomers is affected along collisional events through a basis set superposition error (Boys and Bernardi, 1970) (BSSE) corrected potential energy surface (PES). In addition, the electron density *ρ*(*r*) and its Laplacian ∇^2^*ρ*(*r*) topological analyses were also performed within the atoms in molecules (AIM) theory in order to determine the nature of the intermolecular interactions.

## 2. Methodology

### 2.1. Computational Details

All calculations were performed using the Gaussian09 package (Frisch et al., 2009). The structures were optimized without constraints at MP2(full) method in conjunction with aug-cc-pVTZ-PP for Xe and Rn (Peterson et al., 2003) and aug-cc-pVTZ for the remaining atoms (Dunning, 1989; Woon and Dunning Jr, 1993; Wilson et al., 1999). Vibrational frequencies at the same level of theory were also performed in order to ensure that each minimum has only positive frequencies and that each transition state has only a single imaginary frequency, as well as to obtain the zero point vibrational energy (ZPE). The counterpoise method of Boys and Bernardi (1970) was used to correct the BSSE for binding energy.

AIM analysis (Matta and Boyd, 2007) and graphic representations were performed with the AIMALL program (Keith, 2017) using the MP2(full) density (wavefunction) as input as described in the AIM theory (Dobado et al., 1998; Cortés-Guzmán and Bader, 2005).

### 2.2. Overview of the Transition State Theory

The transition state theory (TST)(Truhlar et al., 1996) was developed primarily by Henry Eyring (Eyring, 1935) and Michael Polanyi (Polanyi and Wigner, 1928) between 1928 and 1935. The TST is an improvement over the so-called theory of collisions (Lewis, 1967), and it is widely used to calculate the rate constants of chemical reactions.

The start point of TST is the existence of a transition state (TS) between the reagents and products. Located at the top of the potential energy barrier and it assumes a quasi-equilibrium between reactants and activated transition state complexes. For a bimolecular reaction given by

(1) $$\begin{array}{l} R_{1}+R_{2}⇋TS\to P_{1}+P_{2}. \end{array}$$

The TS is characterized by a single imaginary frequency along the reaction coordinate of the molecular system which is represented here by ${\bar{\nu }}_{1}$. In its turn, the reaction coordinate can be represented by angular changes in bond distances during the chemical reaction (Henkelman et al., 2002).

The equation that determines the reaction rate is known as the Eyring equation, given by

(2) $$\begin{array}{l} k_{\text{rate}}\left(T\right)=\kappa \left(T\right)\frac{k_{B}T}{h}\frac{{\bar{q}}_{m,\text{TS}}^{\;{}^{\circ}}}{q_{m,R_{1}}^{{}^{\circ}}q_{m,R_{2}}^{{}^{\circ}}}N_{A}e^{-E_{b}^{0}/RT}, \end{array}$$

where 0 < κ(*T*) ≤ 1 is the so-called transmission coefficient, *k*_*B*_ is the Boltzmann constant, *h* is the Planck constant, $q_{m}^{\;{}^{\circ}}$ is the standard molar partition function, *N*_*A*_ is the Avogadro constant, *R* is the gas constant and $E_{b}^{0}$ is the barrier energy with zero-point energy correction. In addition, the TS, *R*_1_ and *R*_2_ subscripts stand for the transition state and reagents, respectively. Thus, the rate constant is determined by the parameters that characterize both reagents and the TS.

The general partition function is formed by the product of translational *q*^trans.^, rotational *q*^rot.^, vibrational *q*^vib.^ and electronic *q*^ele.^ partition functions. The translational partition function for a free particle with mass *m* moving along the length dimension *l*_*x*_ can be evaluated by considering that the separation of energy levels is small and that a large number of states are accessible at room temperatures. Therefore, the energy levels should be continuous and the sum contribution of the translational partition function becomes an integral. Which the solution for the three-dimensional case is (Atkins et al., 2013)

(3) $$\begin{array}{l} q^{\text{trans.}}=\frac{{\left(2\pi mk_{B}T\right)}^{3/2}}{h^{3}}l_{x}l_{y}l_{z}. \end{array}$$

Although the system can be excited at normal modes, the energy levels are discrete for the rotational mode. The three degrees of freedom of spatial rotation and the three moments of inertia *I*_*A*_, *I*_*B*_ and *I*_*C*_ must be taken into account for a non-linear molecule (Atkins et al., 2013), thus

(4) $$\begin{array}{l} q^{\text{rot.}}=\frac{{\left(\pi \right)}^{1/2}}{\sigma }{\left(\frac{8\pi^{2}I_{A}I_{B}I_{C}k_{B}T}{h^{2}}\right)}^{3/2}, \end{array}$$

where σ is the so-called number of symmetry. The vibrational mode has reasonably spaced energy which must be taken into account since they are partially occupied. As a consequence, the vibrational partition function is strictly calculated as a sum over the occupied states. In the case of *n* vibrational degrees of freedom, the vibrational partition function is given by the product of *n* partition functions,

(5) $$\begin{array}{l} q^{\text{vib.}}=\overset{n}{\underset{i}{\prod }}\frac{1}{1-e^{-h\nu_{i}/k_{B}T}}, \end{array}$$

where *ν*_*i*_ is each of the fundamental vibrational frequencies. In most cases, only the lowest energy state is occupied and the electronic energies should not contribute considerably to the total partition function (Atkins et al., 2013). A good approximation is to disregard the contributions of the nuclear and electronic spins and to vanish the fundamental energy level for the electronic partition function. Under these considerations the electronic partition function should be equal to unity (Atkins et al., 2013)

(6) $$\begin{array}{l} q^{\text{ele.}}=1. \end{array}$$

On the other hand, the coefficient *κ*(*T*) represents the tunneling effect of the reaction coordinate of the chemical system and it is usually important for light atoms or molecules at low temperatures. Thus, tunneling estimates were made using both Wigner (Polanyi and Wigner, 1928) and Eckart (Eckart, 1930) methods.

The Wigner tunneling correction proposes a parabolic potential,

(7) $$\begin{array}{l} V_{\text{Wigner}}\left(s\right)=E_{b}-\frac{1}{2}m{\left(2\pi {\bar{\nu }}_{1}\right)}^{2}s^{2}, \end{array}$$

where *E*_*b*_ corresponds the energy potential barrier of MEP, ${\bar{\nu }}_{1}$ is the imaginary frequency of transition state and *s* is the coordinate reaction. This implies in a transmission coefficient given by Bell (1959)

(8) $$\begin{array}{l} \kappa_{\text{Wigner}}\left(T\right)=1-\frac{1}{24}{\left(\frac{h{\bar{\nu }}_{1}}{k_{B}T}\right)}^{2}, \end{array}$$

For very low temperatures, the Wigner tunneling effect is not very effective, and for this reason, it was also employed Eckart tunneling correction (Truhlar et al., 1985).

The Eckart tunneling correction uses a potential of the type

(9) $$\begin{array}{l} V\left(x\right)=\frac{Ae^{\alpha x}}{1\;+\;e^{\alpha x}}+\frac{Be^{\alpha x}}{{\left(1\;+\;e^{\alpha x}\right)}^{2}}, \end{array}$$

where α is a parameter described by

(10) $$\begin{array}{l} \alpha^{2}=-\frac{\mu {\left({\bar{\nu }}_{1}\right)}^{2}B}{2E_{b}^{0}\left(E_{b}^{0}\;-\;A\right)} \end{array}$$

and *μ* is the reduced mass of the system. These parameters determine the barrier width. Here it is important to note that the *A* and *B* can be positive, negative or zero. The *A* parameter corresponds to the energy difference *V*(*x* → −∞) and *V*(*x* → +∞), and *B* is a parameter that measures the height of the barrier given by

(11) $$\begin{array}{l} B=2E_{b}^{0}-A+2\sqrt{E_{b}^{0}\left(E_{b}^{0}-A\right)}. \end{array}$$

So the most usual form for the Eckart's potential in the study of reaction rates is (Truhlar et al., 1985)

(12) $$\begin{array}{l} V_{\text{Eckart}}\left(s\right)=\frac{Ae^{\alpha \left(s-s_{0}\right)}}{1\;+\;e^{\alpha \left(s-s_{0}\right)}}+\frac{Be^{\alpha \left(s-s_{0}\right)}}{{\left[1\;+\;e^{\alpha \left(s-s_{0}\right)}\right]}^{2}}, \end{array}$$

where *s* is the coordinate of the reaction and *s*_0_ is the reaction coordinate corresponding to the maximum of the barrier, which is given by

(13) $$\begin{array}{l} s_{0}=-\frac{1}{\alpha }\ln\;\left(\frac{A\;+\;B}{A\;-\;B}\right). \end{array}$$

Finally, the transmission probability (Bell, 1980), obtained through the solution of the Schrödinger equation with Eckart's potential, is expressed by the following equation

(14) $$\begin{array}{l} P_{\text{Eckart}}\left(E\right)=1-\frac{\cosh\left[2\pi \left(k\;-\;\beta \right)\right]\;+\;\cosh\left(2\pi \delta \right)}{\cosh\left[2\pi \left(k\;+\;\beta \right)\right]\;+\;\cosh\left(2\pi \delta \right)}, \end{array}$$

where *k*, *β* and δ depend on ${\bar{\nu }}_{1}$, *A*, *B* and energy (*E*).

The quantum tunneling correction κ(*T*) can thus be calculated from the ratio between the quantum rate *k*_quan._(*T*) and the classical rate *k*_class._(*T*) in which the particles cross the barrier. Thus, the Eckart tunneling correction with transmission coefficient is given by

(15) $$\begin{array}{l} \kappa \left(T\right)=\frac{k_{\text{quan.}}\left(T\right)}{k_{\text{class.}}\left(T\right)}=\frac{e^{E_{b}/k_{B}T}}{k_{B}T}\int_{0}^{\infty }dE\;P_{\text{Eckart}}\left(E\right)e^{-E/k_{B}T}, \end{array}$$

where integration is performed over all possible energies.

## 3. Results and Discussion

### 3.0.1. Geometric Parameters, Interactions and AIM Analysis

**Figure 1:**
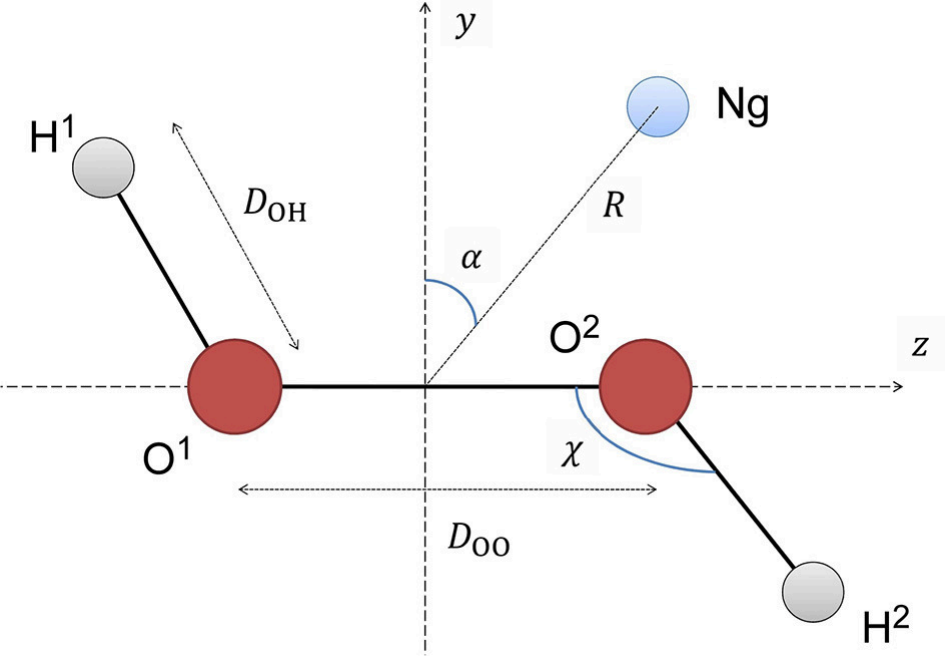
Definitions of the coordinate system used to represent the H_2_O_2_-Ng PES (see text for more details).

The details about the generation of the potential energy surface are described in another work of our group (Roncaratti et al., 2014), so it will be commented briefly here. First, all HP geometry parameters were kept frozen at their equilibrium values of *D*_OO_ = 1.45Å, *D*_OH_ = 0.966Å and the angle HOO = 100.8°. The Ng's position is expressed in terms of the polar coordinates as represented in Figure 1, where *R* is the distance of Ng relative to the middle of O-O bond and α is the polar angle with respect to an axis perpendicular to the O-O bond (*z* axis). The two planes defined by O-O-H atoms are then rotated around the O-O axis, with steps of 1°. In addition, α was equal to 0°, 45°, 90° and *R* distance was varied from 2 to 5Å with steps 0.1Å.

**Figure 2:**
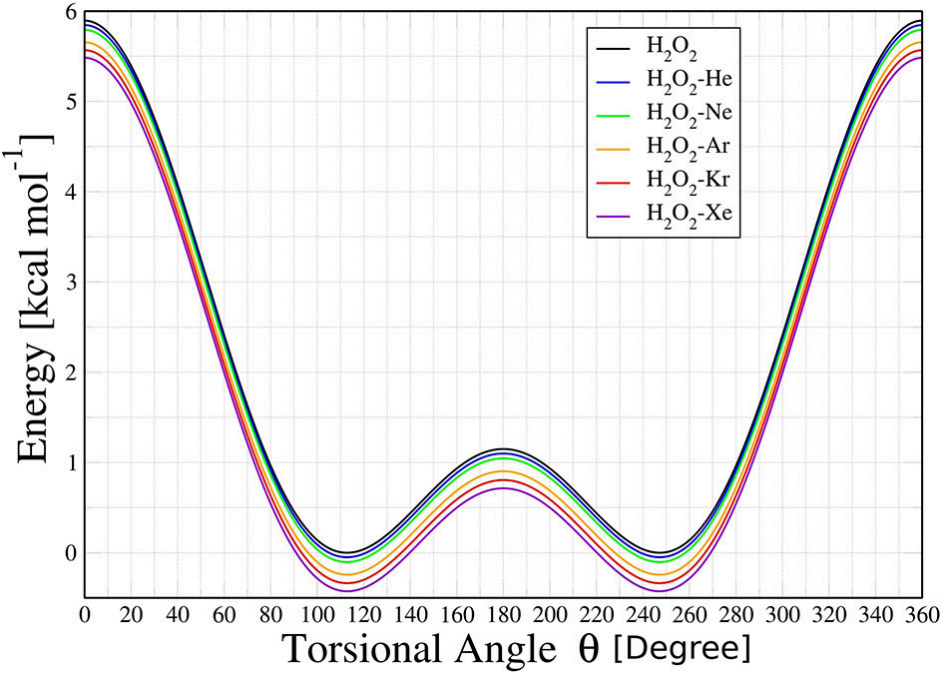
Potential energy curves for H_2_O_2_ and H_2_O_2_-Ng (Ng = He, Ne, Ar, Kr, and Xe) complexes as a function of torsional angle *θ* (in degrees).

Topological studies performed on this adjusted potential energy surface (PES) showed that the HP and HP-Ng complexes have two overall minimum configurations, termed *cis* (labeled as *θ*_−_) and *trans* (labeled as *θ*_+_), separated by two potential barriers, denoted here as *cis*-barrier and *trans*-barrier. The potential energy curves (PEC) obtained from the PES are then presented in Figure 2.

The PECs obtained for HP-Ng complexes are similar in shape and depth. The *cis*-barriers for the HP-Ng complexes are all smaller than the respective value for the free HP. The free HP has a *cis*-barrier of 7.5594 kcal/mol whereas the values for the complexes increase monotonically from 6.9828 kcal/mol for HP-Rn up to 7.5107 kcal/mol for HP-He. In addition the *trans*-barrier values are also lower than the respective value for the free HP, which is 1.0427 kcal/mol, and their values are 1.0928 kcal/mol for HP-He, 1.0817 kcal/mol for HP-Ne, 1.0651 kcal/mol for HP-Ar, 1.0676 kcal/mol for HP-Kr, 1.0736 kcal/mol for HP-Rn and 1.0749 kcal/mol for HP-Xe complexes. For the free HP, the *cis*-barrier and *trans*-barrier experimental energies (Hunt et al., 1965) are 7.0334 ± 0.0715 kcal/mol and 1.1036 ± 0.0114 kcal/mol, respectively. These values are in a good agreement with our results. However, for the HP-Ng complexes we did not find experimental data for comparison.

**Figure 3:**
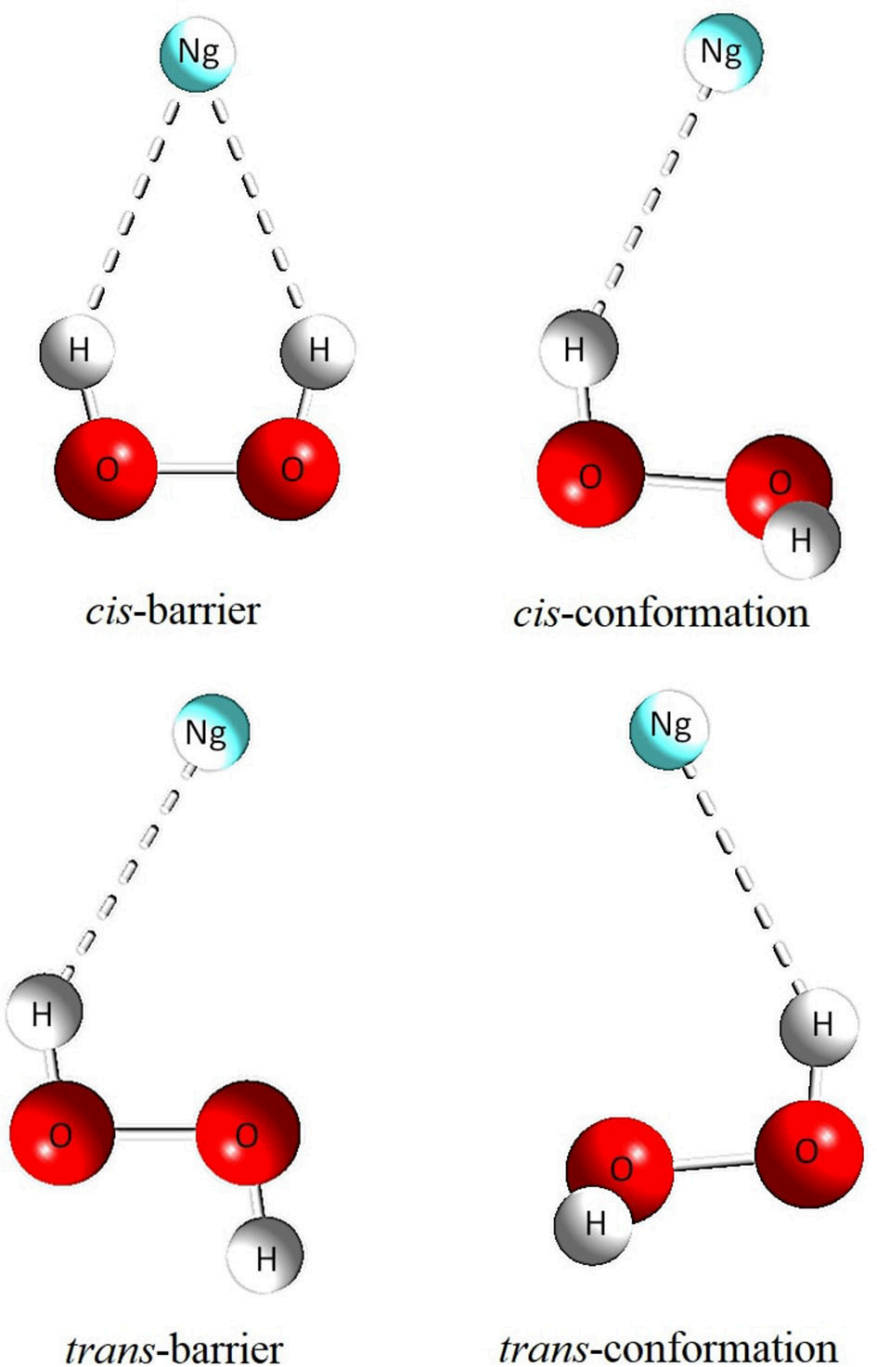
Geometric representation of the *cis*-barrier, *θ*_−_, *trans*-barrier and *θ*_+_ structures.

**Figure 4:**
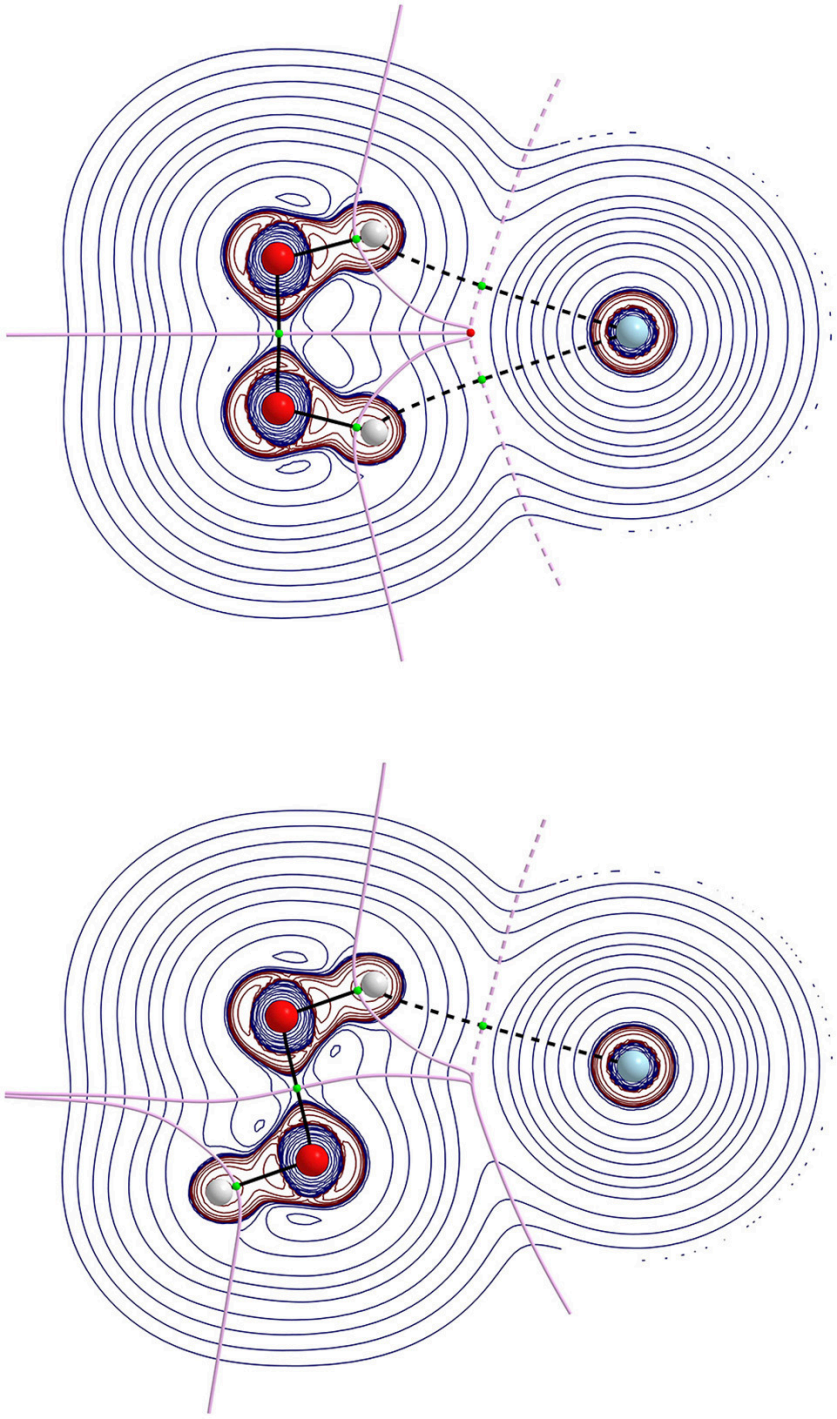
Laplacian of the electron density, ∇^2^*ρ*, contours map in the molecular plane for *cis*-barrier (top) and *trans*-barrier (bottom) conformations of HP-Ne at MP2(full)/aug-cc-pVTZ level. The thick solid lines represent the molecular graph that joints the nuclei, the bond critical point and ring critical point, and also represent the zero flux surface.

The results concerning geometric parameters, interactions and their characterization are summarized in Tables 1–3. The geometrical parameters obtained at MP2(full)/aug-cc-pVTZ, optimized without any constraints for two minimum structures and transition states (presented as *cis* and *trans* barriers) are given in Table 1 together with the graphical representation in Figure 3. Table 2 lists the binding energies corrected and uncorrected for the BSSE. Table 3 shows the numerical results for AIM analysis and Figure 4 depicts the ∇^2^*ρ*(*r*) contour plots for *cis* and *trans* barrier configurations.

**Table 1:** Geometrical parameters (in Å and degree) obtained at MP2(full)/aug-cc-pVTZ level for isolated HP and HP-Ng (Ng=He, Ne, Ar, and Kr) complexes and MP2(full)/aug-cc-pVTZ-PP level for HP-Ng (Ng=Xe and Rn) complexes.

	**HP**	**HP-He**	**HP-Ne**	**HP-Ar**	**HP-Kr**	**HP-Xe**	**HP-Rn**
***cis*****-barrier**							
O-O	1.4570	1.4568	1.4568	1.4566	1.4566	1.4566	1.4567
O-H	0.9641	0.9641	0.9641	0.9644	0.9647	0.9652	0.9654
Ng-HO	-	2.5712	2.6819	2.7932	2.8856	3.0340	3.0930
∠O-O-H	104.2159	104.1917	104.1643	104.0088	103.9379	103.8664	103.8273
∠Ng-H-O	-	143.7666	144.7561	145.8957	146.5895	147.6918	148.1087
∠H-O-O-H	0.0000	0.0000	0.0000	0.0000	0.0000	0.000	0.0000
**θ_−_**							
O-O	1.4478	1.4478	1.4477	1.4478	1.4479	1.4479	1.4479
O-H	0.9641	0.9641	0.9641	0.9645	0.9648	0.9653	0.9656
Ng-HO	-	2.5299	2.6410	2.7361	2.8338	2.9901	3.0506
∠O-O-H	99.7698	99.7484	99.7609	99.6817	99.6684	99.6708	99.6725
∠Ng-H-O	-	148.5851	149.1316	151.3108	152.5323	153.3889	153.4619
∠H-O-O-H	112.5091	112.6401	112.6131	112.6964	112.6133	112.4499	112.3769
***trans*****-barrier**							
O-O	1.4578	1.4584	1.4585	1.4586	1.4587	1.4589	1.4590
O-H	0.9632	0.9633	0.9633	0.9637	0.9639	0.9644	0.9646
Ng-HO	-	2.5545	2.6410	2.7619	2.8581	3.0161	3.0775
∠O-O-H	98.1418	98.1251	98.1150	98.0630	98.0376	98.0253	98.0198
∠Ng-H-O	-	140.9618	142.7619	145.7730	147.4343	148.6294	148.8774
∠H-O-O-H	180.0000	180.0000	179.9996	179.9933	180.0000	180.0000	180.0044
**θ_+_**							
O-O	1.4478	1.4478	1.4477	1.4478	1.4479	1.4479	1.4479
O-H	0.9641	0.9641	0.9641	0.9645	0.9648	0.9653	0.9656
Ng-H-O	-	2.5299	2.6410	2.7361	2.8338	2.9901	3.0515
∠O-O-H	99.7698	99.7484	99.7619	99.6817	99.6684	99.6708	99.6704
∠Ng-H-O	-	148.5851	149.1521	151.3108	152.5323	153.3889	153.4538
∠H-O-O-H	-112.5091	-112.6401	-112.6107	-112.6964	-112.6133	-112.4499	-112.3768
**Other work**^(a)^							
**θ_−_**							
O-O	-	1.441	1.441	1.441	-	-	-
O-H	-	0.964	0.964	0.964	-	-	-
Ng-H-O	-	2.576	2.596	2.828	-	-	-
∠O-O-H	-	100.1	100.1	100.1	-	-	-
∠Ng-H-O	-	151.3	155.5	150.9	-	-	-
∠H-O-O-H	-	111.8	111.8	111.9	-	-	-

(a) *Values obtained by Molina et al. (2002) at MP2/6-311+G(3df,2p) level with BSSE corrections*.

**Table 2:** Binding energies (in kcal/mol) of HP−Ng complexes obtained at MP2(full)/aug−cc−pVTZ level for HP−Ng (Ng=He, Ne, Ar, and Kr) and MP2(full)/aug−cc−pVTZ−PP level for HP−Ng (Ng=Xe and Rn)^a^.

	**BSSE**	***D*_e_**	**$D_{\text{e}}^{\text{BSSE}}$**	***D*_0_**	**$D_{0}^{\text{BSSE}}$**	**Other work^(b)^**
**HP−He**						
*cis*	0.10	−0.19	−0.09	0.10	0.20	
*trans*	0.10	−0.19	−0.09	0.10	0.20	−0.04^(b)^
*cis*-barrier	0.12	−0.26	−0.14	0.03	0.15	
*trans*-barrier	0.07	−0.18	−0.11	0.09	0.18	
**HP-Ne**						
*cis*	0.30	−0.47	−0.17	−0.18	0.12	
*trans*	0.30	−0.47	−0.17	−0.18	0.12	−0.10^(a)^^(b)^
*cis*-barrier	0.36	−0.63	−0.27	−0.34	0.02	
*trans*-barrier	0.29	−0.49	−0.20	−0.20	0.09	
**HP-Ar**						
*cis*	0.62	−1.18	−0.56	−0.89	−0.27	
*trans*	0.62	−1.18	−0.56	−0.89	−0.27	−0.38^(b)^
*cis*-barrier	0.78	−1.63	−0.85	−1.34	−0.56	
*trans*-barrier	0.59	−1.19	−0.60	−0.90	−0.31	
**HP-Kr**						
*cis*	1.41	−2.12	−0.71	−1.83	−0.42	
*trans*	1.41	−2.12	−0.71	−1.83	−0.42	
*cis*-barrier	1.79	−2.89	−1.10	−2.60	−0.81	
*trans*-barrier	1.39	−2.15	−0.76	−1.86	−0.47	
**HP-Xe**						
*cis*	1.23	−2.11	−0.88	−1.82	−0.59	
*trans*	1.23	−2.11	−0.88	−1.82	−0.59	
*cis*-barrier	1.64	−3.04	−1.40	−2.75	−1.11	
*trans*-barrier	1.23	−2.14	−0.91	−1.85	−0.62	
**HP-Rn**						
*cis*	1.63	−2.58	−0.95	−2.29	−0.66	
*trans*	1.63	−2.58	−0.95	−2.29	−0.66	
*cis*-barrier	2.23	−3.77	−1.58	−3.48	−1.25	
*trans*-barrier	1.65	−2.64	−0.99	−2.35	−0.70	

a *Where D_e_ is the electronic binding energy, D_0_=D_e_+ZPE is the electronic binding energy with the zero point energy ZPE, $D_{\text{e}}^{\text{BSSE}}$=D_e_+BSSE and $D_{0}^{\text{BSSE}}$=D_0_+BSSE are the electronic binding energies with BSSE correction*.

(b) *Values obtained by Molina et al. (2002) at MP2/6−311+G(3df,2p) level, for some complexes, with BSSE corrections*.

**Table 3:** Bond critical point (BCP) data for charge density *ρ* (in $\times 10^{-3}e/a_{0}^{3}$), Laplacian of the charge density ∇^2^*ρ* (in $\times 10^{-2}e/a_{0}^{5}$), electronic energy density *H*(*r*) and ellipticity ε for configurations 1(*cis*), 2(*cis*-barrier), 3(*trans*) and 4(*trans*-barrier) of the HP-Ng complexes.

**Complexes**	**Configuration**	**Description**	***ρ***	****∇^2^*ρ*****	**H(r)**	**ε**
He−HP	1	(3,−1) He…H	2.1	1.12	0.0007	0.448
		(3,+1) ring	2.0	1.11	0.0007	
	2	(3,−1) He…H	2.1	1.12	0.0008	0.0965
	3	(3,−1) He…H	2.0	1.10	0.0008	0.2306
	4	(3,−1) He…H	2.1	1.12	0.0008	0.0965
Ne−HP	1	(3,−1) Ne…H	3.0	1.48	0.0007	0.2816
		(3,+1) ring	2.6	1.48	0.0007	
	2	(3,−1) Ne…H	2.9	1.51	0.0008	0.1397
	3	(3,−1) Ne…H	3.0	1.54	0.0008	0.1397
	4	(3,−1) Ne…H	2.9	1.51	0.0008	0.0738
Ar−HP	1	(3,−1) Ar…H	5.7	2.20	0.0010	0.1786
		(3,+1) ring	4.8	2.02	0.0009	
	2	(3,−1) Ar…H	5.9	2.36	0.0012	0.0179
	3	(3,−1) Ar…H	5.6	2.24	0.0012	0.0439
	4	(3,−1) Ar…H	5.9	2.36	0.0012	0.0179
Kr−HP	1	(3,−1) Kr…H	6.44	2.22	0.0009	0.1628
		(3,−1) ring	5.35	2.08	0.0008	
	2	(3,−1) Kr…H	6.63	2.34	0.0010	0.0124
	3	(3,−1) Kr…H	6.35	2.24	0.0010	0.0285
	4	(3,−1) Kr…H	6.63	2.34	0.0010	0.0285
Xe−HP	1	(3,−1) Xe…H	7.0	2.02	0.0006	0.1460
		(3,−1) ring	5.7	1.99	0.0006	
	2	(3,−1) Xe…H	7.0	2.09	0.0007	0.0095
	3	(3,−1) Xe…H	6.7	2.00	0.0007	0.0178
	4	(3,−1) Xe…H	7.0	2.09	0.0007	0.0095
Rn−HP	1	(3,−1) Rn…H	7.1	1.90	0.0005	0.1417
		(3,−1) ring	5.9	1.91	0.0005	
	2	(3,−1) Rn…H	7.1	1.96	0.0006	0.0105
	3	(3,−1) Rn…H	6.8	1.88	0.0006	0.0126
	4	(3,−1) Rn…H	7.1	1.96	0.0006	0.0103

**Figure 5:**
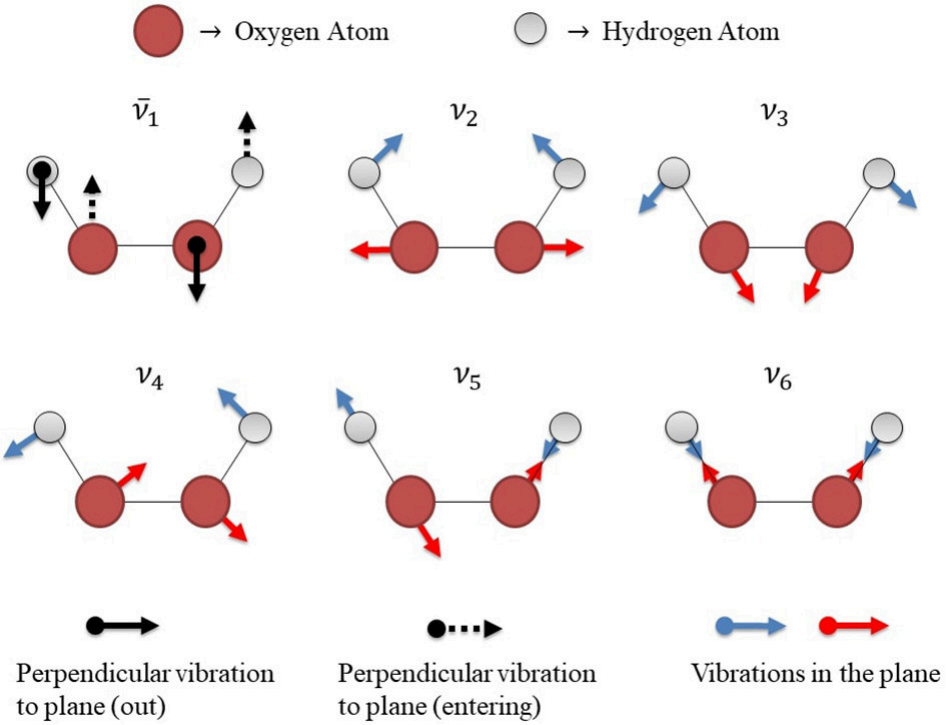
Schematic representation of vibrational modes of the H_2_O_2_ isolated in the transition state. There are 6 vibrational modes, 5 of them correspond to the actual frequencies (ν_2_, ν_3_, ν_4_, ν_5_, and ν_6_) and one of them corresponds to the imaginary frequency ${\bar{\nu }}_{1}$, which represents the frequency along the reaction coordinate.

The PESs yield θ_−_ and θ_+_ as true minima, i.e. without any imaginary frequencies in accordance with results from literature (Maciel et al., 2006; Roncaratti et al., 2014). In addition, all transition state structures displayed a well characterized imaginary frequency around 600 cm^−1^ for *cis* and 400 cm^−1^ for *trans* barriers (see Supplementary Information for further details). Figure 5 describes a schematic representation of vibrational modes of the isolated HP in the transition state with the actual frequencies and an imaginary frequency, which represents the frequency along the reaction coordinate.

For the HP-Ng complexes, the geometrical parameters are almost the same when compared with isolated HP in agreement with the weak interaction of these systems. The HP-Ng distances increase from He up Rn. On average, they are close to 2.55Å(He), 2.65Å(Ne), 2.75Å(Ar), 2.85Å(Kr), 3.00Å(Xe), and 3.06Å(Rn).

Regarding the binding energies, HP-He and HP-Ne are all repulsive, being less repulsive for the *cis* barrier configuration. This can be understood as a consequence of the fact that the noble gases turn out to be the hardest elements (Furtado et al., 2015) and this hardness decreases when the Ng atomic number is increased (the hardness in this context is a resistance to changes in its electronic population Furtado et al., 2015 coupled to Ng's high electronegativity Allen and Huheey, 1980). Although the BSSE increases monotonically from He to Rn, the binding energies also become more attractive as the atomic number increases.

For the four structures of each HP-Ng, the higher binding values are always observed for the *cis*-barrier configuration. As it will pointed latter, the decrease of the rate through the two barriers are not correlated with the binding energy, suggesting the hyperconjugation effects on HP may be important for the decrease of the interaction rate.

Regarding the AIM analysis, the existence of (3,−1) bond critical point (BCP) and its associated atomic interaction line indicates that electronic charge density is accumulated between the linked nuclei (Bader, 1991). In its turn, the values of the charge density *ρ*(*r*) in BCP are small while their corresponding ∇^2^*ρ*(*r*) are positive in accordance with a closed shell type of interaction. As a consequence, all configurations of all complexes show an interaction of a van der Waals type. Since higher ellipticity suggests conjugation and hyperconjugation effects of electron delocalization, these effects seem more pronounced in the HP-He and HP-Ne complexes. Another interesting feature is that all *cis*-barrier configurations of all complexes show a (3,+1) BCP indicating a cyclic nature.

### 3.0.2. Thermal Chiral Rate Analysis

**Figure 6:**
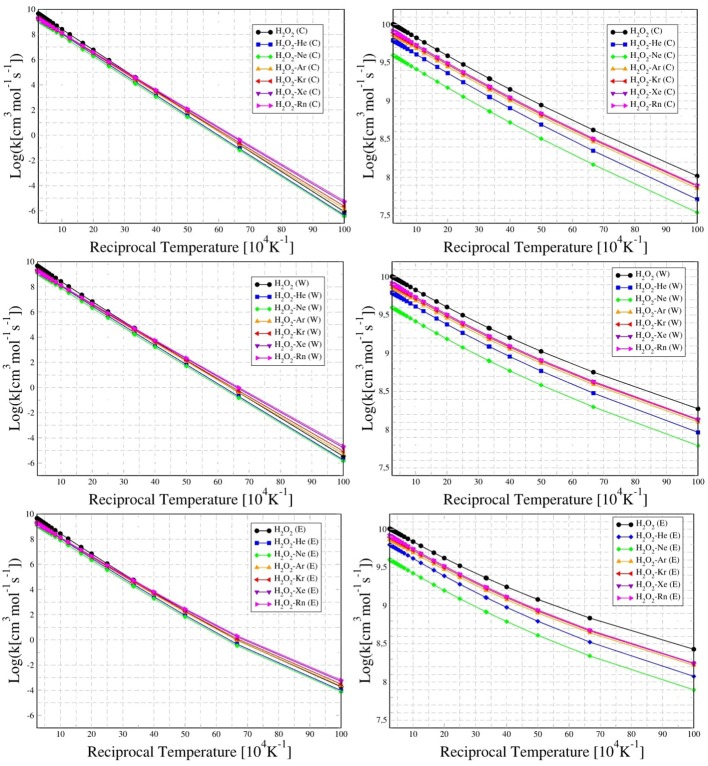
Temperature dependence (from 100 K up to 4,000 K) of the rate constant for conventional (C), Wigner (W), and Eckart (E) tunneling corrections, for *cis* to *trans* (left column) and *trans* to *cis* (right column) chiral conformations of H_2_O_2_ and H_2_O_2_-Ng complexes.

The temperature dependence of the rate constant for *cis* to *trans* (i.e., through *trans*-barrier) and *trans* to *cis* (i.e., through *cis*-barrier) conformations for HP and HP-Ng complexes are presented in Figure 6. These results, in addition to conventional rate, are also exhibited with Eckart's or Wigner's tunneling corrections.

It was found that for the entire 100 K up to 4,000 K range the HP-Ne has the lowest rates for both barriers among all noble gas complexes, followed by HP-He. This result suggests that Ne and He are the noble gases more suitable for study the oriented collision dynamics with HP. In fact, the decrease of CRT shows an inverse correlation with respect the average valence electron energy (Allen, 1989), which follows the sequence (from higher to lower values): Ne, He, Ar, Kr; with Xe and Rn having very close values.

Nevertheless, there is a trend of rate increase as are move from Ar up to Rn. It is interesting to note that although this behavior is very similar regarding the *cis*-barrier for all rates (conventional, Wigner e Eckart), it seems that the tunneling is more important to describe the *trans* barrier's rate, where there is a significant difference for Eckart's values specially in the 100–200 K range when compared to respective Wigner and conventional results.

The final thermal rate constant can be expressed in the two familiar Arrhenius forms. In this work, the first is the Arrhenius modified form given by

(16) $$\begin{array}{l} k\left(T\right)=AT^{n}e^{-E_{a}/RT}, \end{array}$$

where *A* is the pre-exponential factor, *T* a temperature, *n* is a real number, R is the universal gas constant and *E*_*a*_ is the activation energy. The second is the d-Arrhenius form (Aquilanti et al., 2010; Silva et al., 2013; Carvalho-Silva et al., 2017) expressed by

(17) $$\begin{array}{l} k\left(T\right)=A{\left(1-d\frac{E_{a}}{RT}\right)}^{1/d} \end{array}$$

where *d* is a parameter that yield the degree of deformation of the exponential function.

The curve obtained by the reaction rate constant vs. the temperature can be fitted (Ramalho et al., 2011) to obtain the parameters *A*, *n* and *E*_*a*_ for the Arrhenius modified form, as presented in Table 4, and the parameters *A*, *d*, and *E*_*a*_ for d-Arrhenius form, as presented in Table 5. This feature confirms the trend of lower *k*(*T*) observed for HP-Ng complexes for both barriers when compared to isolated HP.

**Table 4:** Adjusted parameters for the modified Arrhenius equation for conventional (C), Wigner (W) and Eckart (E) models with *E*_*a*_ in kcal/mol.

**Molecule**	***A*(C)**	***A*(W)**	***A*(E)**	***n*(C)**	***n*(W)**	***n*(E)**	***E*_*a*_(C)**	***E*_*a*_(W)**	***E*_*a*_(E)**
***CIS*****-BARRIER**									
HP	6.1018×10^9^	2.8802×10^9^	11.0401×10^7^	0.0780	0.1650	0.5572	7.3552	7.0124	6.1979
HP-He	2.6279×10^9^	1.2418×10^9^	4.8886×10^7^	0.0759	0.1627	0.5517	7.3155	6.9737	6.1659
HP-Ne	1.7572×10^9^	0.8310×10^9^	3.3715×10^7^	0.0755	0.1622	0.5476	7.2742	6.9333	6.1322
HP-Ar	2.6503×10^9^	1.2611×10^9^	6.2821×10^7^	0.0732	0.1593	0.5200	7.0818	6.7464	5.9960
HP-Kr	2.5508×10^9^	1.2177×10^9^	6.7090×10^7^	0.0726	0.1584	0.5070	6.9795	6.6470	5.9211
HP-Xe	2.4190×10^9^	1.1593×10^9^	7.2082×10^7^	0.0721	0.1574	0.4915	6.8421	6.5129	5.8164
HP-Rn	2.3730×10^9^	1.1396×10^9^	7.5261×10^7^	0.0720	0.1571	0.4839	6.7739	6.4465	5.7646
***TRANS*****-BARRIER**									
HP	6.1504×10^9^	4.1827×10^9^	3.6443×10^9^	0.0758	0.1212	0.1376	0.9082	0.7686	0.6923
HP-He	3.7940×10^9^	2.5942×10^9^	2.3349×10^9^	0.0764	0.1212	0.1340	0.9519	0.8145	0.7657
HP-Ne	2.4228×10^9^	1.6587×10^9^	1.5061×10^9^	0.0760	0.1207	0.1324	0.9413	0.8044	0.7575
HP-Ar	4.4132×10^9^	3.0523×10^9^	2.7009×10^9^	0.0775	0.1210	0.1357	0.9178	0.7851	0.7296
HP-Kr	4.6795×10^9^	3.2492×10^9^	2.8867×10^9^	0.0784	0.1214	0.1356	0.9177	0.7866	0.7325
HP-Xe	4.8857×10^9^	3.4004×10^9^	3.0393×10^9^	0.0791	0.1218	0.1354	0.9217	0.7915	0.7398
HP-Rn	4.9038×10^9^	3.4166×10^9^	3.0635×10^9^	0.0793	0.1218	0.1350	0.9227	0.7930	0.7424

**Table 5:** Adjusted parameters for the d-Arrhenius equation for conventional (C), Wigner (W) and Eckart (E) models with *E*_*a*_ in kcal/mol.

**Molecule**	***A*(C)**	***A*(W)**	***A*(E)**	***d*(C)**	***d*(W)**	***d*(E)**	***E*_*a*_(C)**	***E*_*a*_(W)**	***E*_*a*_(E)**
***CIS*****-BARRIER**									
HP	11.7×10^9^	11.3×10^9^	11.3×10^9^	−0.0013	−0.0025	−0.0143	7.5627	7.4084	8.0939
HP−He	5.0×10^9^	4.8×10^9^	5.7×10^9^	−0.0013	−0.0025	−0.0143	7.5174	7.3641	8.0431
HP−Ne	3.3×10^9^	3.2×10^9^	3.8×10^9^	−0.0013	−0.0025	−0.0143	7.4753	7.3227	7.9943
HP−Ar	4.9×10^9^	4.7×10^9^	5.5×10^9^	−0.0013	−0.0026	−0.0142	7.2768	7.1297	7.7525
HP−Kr	4.7×10^9^	4.5×10^9^	5.3×10^9^	−0.0013	−0.0026	−0.0142	7.1726	7.6274	7.6274
HP−Xe	4.4×10^9^	4.3×10^9^	4.9×10^9^	−0.0014	−0.0027	−0.0142	6.8421	6.5129	7.4631
HP−Rn	4.3×10^9^	4.2×10^9^	4.8×10^9^	−0.0014	−0.0028	−0.0143	6.9644	6.8252	7.3822
***TRANS*****-BARRIER**									
HP	11.7×10^9^	11.3×10^9^	11.8×10^9^	−0.0731	−0.1578	−0.2131	1.1387	1.1583	1.1603
HP−He	7.3×10^9^	7.3×10^9^	7.4×10^9^	−0.0731	−0.1414	−0.1796	1.1822	1.1992	1.2210
HP−Ne	4.6×10^9^	4.6×10^9^	4.7×10^9^	−0.0743	−0.1441	−0.1816	1.1705	1.1879	1.2079
HP−Ar	8.5×10^9^	8.6×10^9^	8.6×10^9^	−0.0791	−0.1510	−0.1959	1.1520	1.1715	1.1928
HP−Kr	9.1×10^9^	9.1×10^9^	9.2×10^9^	−0.0798	−0.0026	−0.1944	1.1545	1.1743	1.1950
HP−Xe	9.6×10^9^	9.6×10^9^	9.7×10^9^	−0.0797	−0.1497	−0.1909	1.1605	1.1805	1.2007
HP−Rn	9.6×10^9^	9.6×10^9^	9.7×10^9^	−0.0796	−0.1491	−0.1894	1.1619	1.1818	1.2017

It can be also observed in Figure 6 that the *trans* to *cis* conformation rate of HP is lower (in the range 100–200 K) than the corresponding ones for HP-Ar, HP-Kr, HP-Xe, and HP-Rn. In the case of the chiral transition from *cis* to *trans*, the rates of all HP-Ng complexes are lower than that of the isolated HP. These results showed that the transition rate from *cis* to *trans* is greater than the corresponding *trans* to *cis* for both the isolated HP molecule and for all HP-Ng complexes. This suggests that the most important barrier that separates the chiral configurations of the isolated HP and the HP-Ng complexes is the *trans*-barrier, since it is the smallest. The energy of the HP's *trans*-barrier is relatively small (1.0427 kcal/mol) compared to its *cis*-barrier (7.5595 kcal/mol) as already seen in Figure 2.

**Figure 7:**
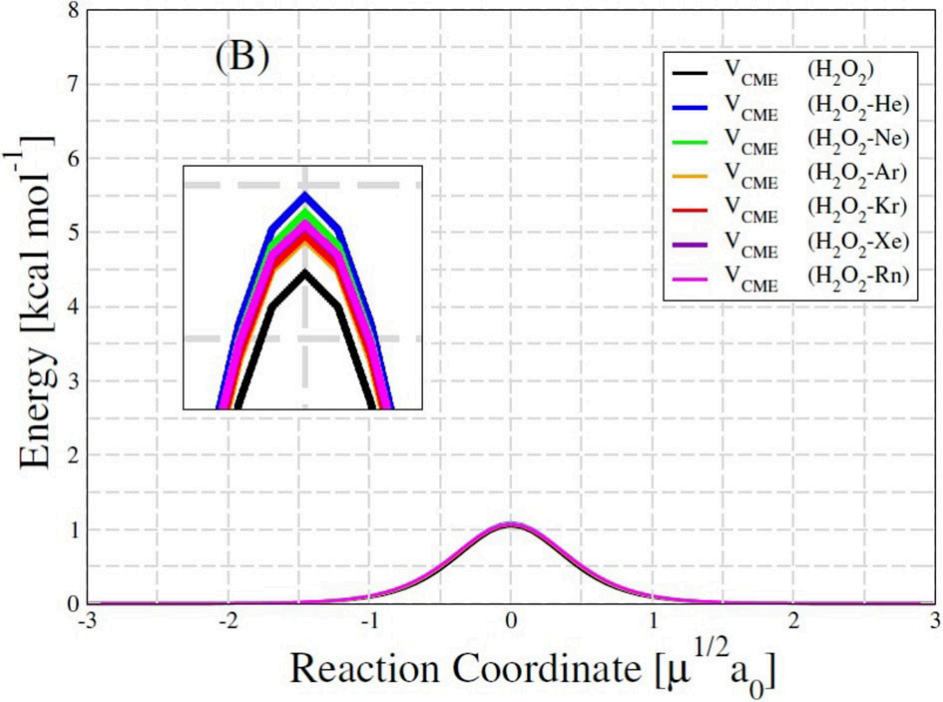
*Trans*-barrier (B) energies of HP and H_2_O_2_-Ng complexes.

An interesting result is presented in the Table 6. Although the increase in the *trans*-barrier of the HP-Ng complexes relative to HP is considerably small (see Figure 7), the change in the transition rate from *cis* to *trans* is relatively high. This is verified for high (4,000 K), room (298.15 K) and also for low temperatures (100 K). The most pronounced decrease in the rate corresponds to the HP-Ne complex, in which the decrease of the *trans*-barrier of just 0.0389 kcal/mol (see Figure 6) corresponds to a decrease of over 60% for the *cis*-*trans* transition rate, followed by HP-He. It is also interesting to note that this small change in energy barrier but with a substantial change in rate was also observed for other HP-Ng complexes. For example, HP-Ar complex showed a decrease of just 0.0224 kcal/mol but a 28.68% decrease of rate at 100 K.

**Table 6:** Difference between heights of *trans*-barrier of HP and HP-Ng complexes and relative decrease of the transition rate of *cis* to *trans* configuration for representative temperatures (4, 000, 298.15, and 100 K).

		**Decrease**	**Decrease**	**Decrease**
**Molecule**	**Δ(kcal/mol) ^(a)^**	**of rate (%)**	**of rate (%)**	**of rate (%)**
		**(*T* = 4, 000 K)**	**(*T* = 298.15 K)**	**(*T* = 100 K)**
HP	0	0	0	0
HP-He	0.0501	50.39	42.48	38.32
HP-Ne	0.0389	66.66	62.66	60.67
HP-Ar	0.0224	31.07	28.68	27.26
HP-Kr	0.0250	26.50	24.02	22.34
HP-Xe	0.0310	24.45	20.90	18.47
HP-Rn	0.0322	24.53	20.70	18.10

(a) *Δ = E_b-trans_(HP)−E_b-trans_(HP-Ng)*.

Finally, at a temperature close to 300K, the Boltzmann distribution shows that about 16% of HP's population has higher energy than the *trans*-barrier with thermal fluctuations of approximately 1.7686 kcal/mol (Ball and Brindley, 2016). It has also been found that at low temperatures the chiral interconversion quantum encapsulation time of HP is very small. At a temperature of 100 K this time is <1 pico-second (Bitencourt et al., 2008), and at temperatures close to 0 K which can reach 3 pico-seconds.

## 4. Conclusions

The obtained results indicate that the chiral transition rate of *trans* to *cis* configuration of hydrogen peroxide in the presence of the noble gases He and Ne were the lowest over the entire temperature range of 4,000–100 K.

The AIM analysis shows that the interaction between H_2_O_2_ and the noble gases should be a van der Waals type. Although the H_2_O_2_ acts as an acid in the context of this investigation, the high hardness and high electronegativity of the nobles gases hold their electrons very tight to permit a covalence bond between H_2_O_2_ and Ng. On the other hand, it seems that both He and Ne are better able to affect the hyperconjugation effect and destabilizing repulsion among the lone pairs that are responsible for rotational barriers (Song et al., 2005). This may explain why the chiral transition rate decreases more for the complexes composed by Ne and He atoms, the hardest and more electronegative noble gases (Furtado et al., 2015).

Finally, the *trans*-barrier plays an important role because it is much smaller than the *cis*-barrier. The results showed that a small increase in the *trans*-barrier height in the complexes is responsible for a significant decrease in the rate of transition from *cis* to *trans*. Thus, these effects may contribute to the feasibility of separating one or the other enantiomer of the H_2_O_2_ molecule.

## Author Contributions

RG conceived and supervised the study. RG also helped write the paper. YS performed the H_2_O_2_-Ng electronic and thermal chiral rate calculations. PN determined the H_2_O_2_-Ng minimum and transition state configurations and LdM used the AIM theory to perform the H_2_O_2_-Ng topological analyses and wrote the manuscript, which was reviewed by all authors.

## Acknowledgments

We gratefully acknowledge the financial support from the Brazilian Research Councils CNPq and FAPDF.

### Conflict of Interest Statement

The authors declare that the research was conducted in the absence of any commercial or financial relationships that could be construed as a potential conflict of interest.
