# Supplementary material for: Theoretical Investigation on H2O2-Ng (He, Ne, Ar, Kr, Xe, and Rn) Complexes Suitable for Stereodynamics: Interactions and Thermal Chiral Rate Consequences
Source: Front Chem. 2019 Jan 18;6:671. doi: 10.3389/fchem.2018.00671 (PMC6345723; doi:10.3389/fchem.2018.00671)
Supplement: Supplementary file 1 [file Data_Sheet_1.pdf]

Theoretical investigation on H_2O_2-Ng (He, Ne, Ar, Kr, Xe and Rn) complexes suitable for stereodynamics: interactions and thermal chiral rate consequences

Yuri Alves de Oliveira Só,[†] Pedro Henrique de Oliveira Neto,[†] Luiz Guilherme Machado de Macedo,[‡] and Ricardo Gargano[*,†]

†*Institute of Physics, University of Brasília, Brasília, 70.919-970, Brazil*

‡*Faculty of Biotechnology, Federal University of Pará, Pará, Brazil*

E-mail: gargano@unb.br

Abstract

Although molecular collisions of noble gases (Ng) can be theoretically used to distinguish between the enantiomers of hydrogen peroxide - H_2O_2 (HP), little is known about the effects of Ng-HP interactions on the chiral rate. In this work, the chiral rate as a function of temperature (CRT) between enantiomeric conformations of HP and Ng (Ng=He, Ne, Ar, Kr, Xe, and Rn) are presented at MP2(full)/aug-cc-pVTZ level of theory through a fully basis set superposition error (BSSE) corrected potential energy surface. The results show that: (a) the CRT is highly affected even at a small decrease in the height of trans-barrier; (b) its smallest values occurs with Ne for all temperatures between 100 and 4000K; (c) that the decrease of CRT shows an inverse correlation with respect to the average valence electron energy of the Ng and (d) Ne and He may be the noble gases more suitable for study the oriented collision dynamics

of HP. In addition to binding energies, the electron density ρ and its Laplacian $\nabla^2\rho$ topological analyses were also performed within the atoms in molecules (AIM) theory in order to determine the nature of the HP-Ng interactions. The results of this work provide a more complete foundation on experiments to study HP's chirality using Ng in crossed molecular beams without a light source.

Table 1: Vibrational frequencies for the *cis* and *trans* conformation for HP and HP-Ng complexes.

ν_i (cm^{-1})	HP	HP-He	HP-Ne	HP-Ar	HP-Kr	HP-Xe	HP-Rn
cis							
ν_1	387.9004	387.9605	387.7946	390.1990	391.9552	393.2807	393.5368
ν_2	932.6970	932.6257	933.0004	932.3813	932.1304	931.7477	931.4836
ν_3	1317.9703	1318.0590	1318.2160	1318.8125	1319.3422	1319.9016	1320.0493
ν_4	1425.0663	1425.5286	1425.5803	1426.0879	1426.1251	1425.8504	1425.5990
ν_5	3809.6464	3809.4908	3809.6305	3804.6301	3797.8432	3787.3547	3781.1833
ν_6	3809.6781	3811.2516	3811.2786	3808.7514	3808.2951	3807.6651	3807.3341
ν_7	-	25.1840	19.6476	35.1826	34.2194	34.1822	33.2499
ν_8	-	32.6939	29.0404	62.6122	65.1551	67.5781	67.6767
ν_9	-	49.3118	39.4196	66.8164	77.2847	84.1395	86.4779
trans							
ν_1	387.9004	387.9605	387.7915	390.1991	391.9548	393.2808	393.5755
ν_2	932.6970	932.6257	933.0073	932.3813	932.1304	931.7477	931.4465
ν_3	1317.9703	1318.0590	1318.2229	1318.8125	1319.3422	1319.9016	1320.0412
ν_4	1425.0663	1425.5286	1425.5781	1426.0879	1426.1250	1425.8504	1425.5887
ν_5	3809.6464	3809.4908	3809.6296	3804.6301	3797.8430	3787.3547	3781.1753
ν_6	3809.6781	3811.2516	3811.2632	3808.7514	3808.2950	3807.6651	3807.3096
ν_7	-	25.1840	19.8435	35.1826	34.2197	34.1822	33.2528
ν_8	-	32.6939	29.0001	62.6122	65.1555	67.5781	67.5979
ν_9	-	49.3118	39.4354	66.8163	77.2821	84.1395	86.6424

Table 2: Vibrational frequencies of *cis*-barrier and *trans*-barrier for HP and HP-Ng complexes.

ν_i (cm^{-1})	HP	HP-He	HP-Ne	HP-Ar	HP-Kr	HP-Xe	HP-Rn
cis-barrier							
$\bar{\nu}_1$	-598.2946	-596.7185	-595.4821	-586.6731	-582.2256	-576.9669	-574.2600
ν_2	922.3430	922.9026	922.8458	923.1242	922.9421	922.5264	922.1289
ν_3	1345.6124	1346.3719	1346.7771	1348.4733	1349.5248	1350.3115	1350.4260
ν_4	1434.4059	1435.0125	1435.3437	1436.7261	1437.5447	1438.3677	1438.6176
ν_5	3791.7867	3794.2746	3794.1854	3789.6965	3784.2555	3775.1637	3769.4642
ν_6	3830.5039	3831.1446	3831.4581	3824.1636	3817.5545	3807.0149	3801.1354
ν_7	-	39.6614	38.9830	56.4781	54.3728	54.9297	53.8764
ν_8	-	41.5825	40.6905	61.8047	67.1019	71.5483	72.1307
ν_9	-	55.4464	47.9603	95.2621	110.6184	122.5549	127.1160
trans-barrier							
$\bar{\nu}_1$	-305.3814	-302.2509	-301.5356	-295.6440	-293.3837	-292.0233	-291.4070
ν_2	933.5295	931.6392	931.5626	930.9389	930.5606	930.0158	929.6924
ν_3	1247.2248	1247.2087	1247.3852	1247.9772	1248.3096	1248.4804	1248.4890
ν_4	1520.9784	1520.2910	1520.3196	1519.7631	1519.5856	1519.1821	1518.8958
ν_5	3829.9970	3829.6102	3829.5481	3825.1959	3820.3606	3811.7545	3806.3420
ν_6	3839.9068	3839.6107	3839.5002	3835.2261	3833.1093	3831.1807	3830.3365
ν_7	-	32.2986	30.1923	41.9701	39.3565	38.5814	37.5190
ν_8	-	35.2457	39.4511	60.1563	62.7145	64.8172	64.8815
ν_9	-	50.4281	40.9030	72.0319	81.8674	87.9305	90.3954

Table 3: Total energies (in a.u.) for *cis* and *trans* transitions state, and *cis* e *trans* conformations.

Molecule	$E_{\text{TS-}cis}$	E_{cis}	$E_{\text{TS-}trans}$	E_{trans}
HP	-151.36543	-151.37748	-151.37572	-151.37748
HP-He	-154.26047	-154.27244	-154.27069	-154.27244
HP-Ne	-280.18504	-280.19694	-280.19522	-280.19694
HP-Ar	-678.42417	-678.43576	-678.43406	-678.43576
HP-Kr	-2903.70593	-2903.71736	-2903.71566	-2903.71736
HP-Xe	-479.91676	-479.92799	-479.92628	-479.92799
H_2O_2-Rn	-439.17367	-439.18479	-439.18308	-439.18479

Table 4: *Cis*-barrier ($E_{b\text{-}cis}$) and *trans*-barrier ($E_{b\text{-}trans}$) energy values (in kcal/mol) and equivalent temperature (in K) for HP and HP-Ng complexes.

Molecule	E_{cis-b} (kcal/mol)	T_{cis-b}(K)	$E_{b\text{-}trans}$ (kcal/mol)	$T_{b\text{-}trans}$ (K)
H_2O_2	7.5595	3804.1	1.0427	524.7
H_2O_2-He	7.5106	3779.5	1.0928	549.9
H_2O_2-Ne	7.4683	3758.2	1.0816	544.3
H_2O_2-Ar	7.2731	3660.0	1.0651	536.0
H_2O_2-Kr	7.1744	3610.3	1.0677	537.3
H_2O_2-Xe	7.0452	3545.3	1.0737	540.3
H_2O_2-Rn	6.9828	3513.9	1.0749	540.9
